# A granular activated carbon/electrochemical hybrid system for onsite treatment and reuse of blackwater

**DOI:** 10.1016/j.watres.2018.07.070

**Published:** 2018-11-01

**Authors:** Tate W. Rogers, Tess S. Rogers, Mikayla H. Stoner, Katelyn L. Sellgren, Brendon J. Lynch, Aaron A. Forbis-Stokes, Brian R. Stoner, Brian T. Hawkins

**Affiliations:** a Triangle Environmental Health Initiative, Durham, NC, USA; b RTI International, Research Triangle Park, NC, USA; c Department of Electrical and Computer Engineering, Duke University, Durham, NC, USA; d Center for WaSH-AID, Duke University, Durham, NC, USA; e Department of Civil and Environmental Engineering, Duke University, Durham, NC, USA; f Biomass Controls, Durham, NC, USA

**Keywords:** Sanitation, Water reuse, Activated carbon, Chemical oxygen demand, Electrochemical disinfection

## Abstract

Over 1/3 of the global population lacks access to improved sanitation, leading to disease, death, and impaired economic development. Our group is working to develop rapidly deployable, cost-effective, and sustainable solutions to this global problem that do not require significant investments in infrastructure. Previously, we demonstrated the feasibility of a toilet system that recycles blackwater for onsite reuse as flush water, in which the blackwater is electrochemically treated to remove pathogens due to fecal contamination. However, this process requires considerable energy (48–93 kJ/L) to achieve complete disinfection of the process liquid, and the disinfected liquid retains color and chemical oxygen demand (COD) in excess of local discharge standards, negatively impacting user acceptability. Granular activated carbon (GAC) efficiently reduces COD in concentrated wastewaters. We hypothesized that reduction of COD with GAC prior to electrochemical treatment would both improve disinfection energy efficiency and user acceptability of the treated liquid. Here we describe the development and testing of a hybrid system that combines these technologies and demonstrate its ability to achieve full disinfection with improved energy efficiency and liquid quality more suitable for onsite reuse and/or discharge.

## Highlights

• Recycled blackwater was treated with granular activated carbon to reduce organic load.
• Reducing organic load improved electrochemical disinfection energy efficiency.
• This combination will enable more energy-efficient treatment and reuse of blackwater.

## 1 Introduction

This study is a part of an ongoing project to address the needs of over a third of the world's population that lack access to improved sanitation. Inadequate sanitation leads to the spread of diarrheal diseases, resulting in the death of over 530,000 children under the age of five every year (WHO, 2017), in addition to tremendous economic costs and unrealized economic benefits (Hutton, 2013). Typical centralized water and wastewater infrastructures are prohibitively expensive to build and maintain in developing areas (Dodane et al., 2012). Therefore, novel approaches for affordable and reliable on-site treatment of human waste are essential to combat these negative health and economic impacts.

Our team, led by the Duke University Center for Water, Sanitation, Hygiene and Infectious Disease (WaSH-AID) is developing a toilet that converts human waste into burnable fuel, stored energy, and disinfected water suitable for non-potable reuse and/or discharge. The project's ultimate goal is to operate this unit without piped-in water, a sewer connection, or outside electricity. To deliver a system that can meet these goals, the liquid must be treated to at least surface discharge standards and must be aesthetically acceptable for the intended reuse purpose.

Our liquid treatment system utilizes a solids separation mechanism and settling tanks to achieve up to 86% removal of total suspended solids (TSS) and an electrochemical process that presently requires 48–93 kJ/L (13–26 kWh/m^3^) to achieve complete disinfection (Hawkins et al., 2017, Hawkins et al., 2018, Sellgren et al., 2017). We have hypothesized that the high energy requirement for disinfection is likely attributable to the soluble and suspended chemical oxygen demand (COD) in the process liquid (Hawkins et al., 2018). In addition, the treated liquid maintains sufficient color and odor to require a further “polishing” step; surveys from field testing of a prototype unit at CEPT University in Ahmedabad, India confirmed that users were dissatisfied with color and odor of the disinfected liquid recycled for flushing (Elledge et al., unpublished results.)

This paper focuses on the development and testing of a granular activated carbon (GAC) module to integrate into the liquid treatment system with the goal of mitigating both the energy budget and user acceptability issues. GAC has been widely used in wastewater treatment for removal of organic contaminants (Pollard et al., 1992, Tchobanoglous et al., 2003). The high surface area to volume ratios of the material facilitate high efficiency removal of organic and inorganic compounds through adsorption. Packed bed column filters with GAC as the media allow oxygenation and promote adsorption of inorganics, biological degradation of organic contaminants, and have high resource recovery potential (Forbis-Stokes et al., 2018, Huggins et al., 2016). Here, we demonstrate that combining GAC packed bed column filters with electrochemical disinfection reduces the overall energy requirements (from 70 ± 12 to 20 ± 9 kJ/L for disinfection) and significantly improves water quality parameters critical to achieving user acceptance of the reused liquid in our system.

## 2 Materials and methods

### 2.1 Blackwater production and electrochemical treatment

Blackwater was obtained from a prototype toilet and liquid disinfection system which has been described in detail previously (Hawkins et al., 2017, Sellgren et al., 2017). Procedures for collection of urine and feces from healthy volunteers were approved by the institutional review board at RTI International. Samples were flushed into the prototype toilet (initially charged with tap water for flush liquid) at rates intended to approximate estimated average per person urine and fecal production rates of 1.5 L and 130 g per day, respectively (Rose et al., 2015). Total urine volumes (Σ*v*_*urine*_) and fecal masses (Σ*m*_*feces*_) flushed were therefore used to calculate user-day equivalents (UDE), which were used to index data collected to the estimated usage of the system from startup with tap water:

(1) $$UDE=\frac{\frac{\sum v_{urine}}{1.5L}+\frac{\sum m_{feces}}{130g}}{2}$$

Under normal operation, 30-L batches of blackwater were disinfected by an electrochemical process using a commercially available electrochemical cell (Hayward Salt&Swim 3C) as previously described in detail (Sellgren et al., 2017, Hawkins et al., 2017). Alternatively, untreated blackwater was taken from the system for smaller scale pilot experiments with GAC (see below). Disinfected blackwater was recycled through the system as flush liquid for subsequent flush cycles and excess processed liquid was discharged.

### 2.2 Granular activated carbon

**Fig. 1:**
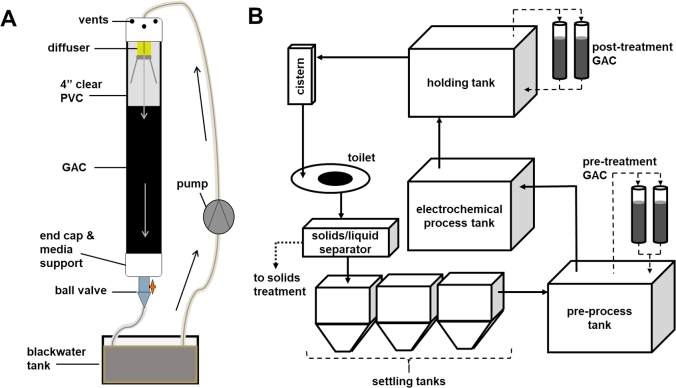
**Experimental systems. A:** GAC packed bed column filter lab setup. **B:** Schematic of GAC packed bed column filters integrated into the liquid treatment system.

Aquacarb^®^ 830, an 8 × 30 mesh-sized GAC derived from bituminous coal, (Evoqua, Pittsburgh, PA) was used for all studies described herein. The apparatus used for bench-scale proof-of-concept studies consisted of a transparent section of PVC pipe, 10.2 cm (4 in) in diameter by 1.23 m (4 ft) in length (Fig. 1A). A 0.95 cm (3/8-in) ball valve was threaded through a 10.2 cm (4 in) end cap to serve as an outlet, and a #40 mesh was affixed inside the cap to support the filter media. The cap (with the valve and mesh) was then affixed to one end of the pipe with PVC cement. The pipe was mounted vertically to a stand with the outlet facing downward and 5 L (∼2.3 kg) of pre-washed GAC were poured into the column. Water was flushed through the column until no fines were observed coming out of the outlet prior to beginning blackwater studies.

For bench-scale proof of concept studies, 8-L batches of blackwater were taken from the prototype liquid treatment system and placed in a reservoir, pumped to the top of the column through a diffuser (to distribute flow across the top surface and aerate) at approximately 120 ml/min. A tube was run from the outlet back to the reservoir, enabling continuous recirculation. The GAC empty-bed residence time (EBRT) was 0.69 h under this pumping regimen. Following at least 24 h GAC treatment, the blackwater was then electrochemically treated by placing an electrochemical cell (the same model used in the prototype system) in the reservoir and applying 24 VDC for up to 90 min. For control (CON) experiments, blackwater was electrochemically treated without being run through the GAC filter.

For studies that integrated GAC treatment into the existing liquid treatment system (Fig. 1B), two columns were constructed out of the same materials as above and each were filled with 8 L (3.6 kg) of GAC. A manifold recirculation circuit was run between the columns and the pre-process tank and the filters were run in the same manner as described above. In addition, overflow outlets were run from the top of the columns back into the pre-process tank. For the final set of studies, an identical system was also added to the post-process holding tank.

### 2.3 Water quality measurements

Conductivity was measured with a Myron L 6PFCE Ultrameter II (Myron L Company, Carlsbad, CA). COD was measured with a HACH DR 900 colorimeter using the Reactor Digestion Method (HACH method 8000) and a HACH DRB200 reactor (HACH, Loveland, CO). Turbidity was measured with a HACH 2100Q IS. Total solids (TS) were determined by the EPA method (EPA, 2001). Total suspended solids (TSS) were determined using Standard Method 2540D. Color was measured with a HACH CO-1 test kit.

### 2.4 Microbial enumeration

Disinfection efficacy was determined by using the most probable number (MPN) method, and the energy required to achieve the desired threshold of disinfection (MPN = 5/ml) was determined by interpolating the plot of log (MPN) versus *E*_*n*_, as previously described (Hawkins et al., 2017).

### 2.5 Data analysis

Electrochemical energy per volume of process liquid used at time *n* (*E*_*n*_) was calculated by:

(2) $$E_{n}=\;\;\frac{\;V\int_{0}^{n}I\left(t\right)dt}{v}$$

where V is the voltage, I is the current through the electrochemical cell measured with a Mastech MS2138R AC/DC clamp meter, and *ν* is the volume being treated. The integral of current with time was estimated by the trapezoid method.

Statistical calculations were performed with GraphPad Prism v7.04.

## 3 Results and discussion

### 3.1 Benchtop study

**Fig. 2:**
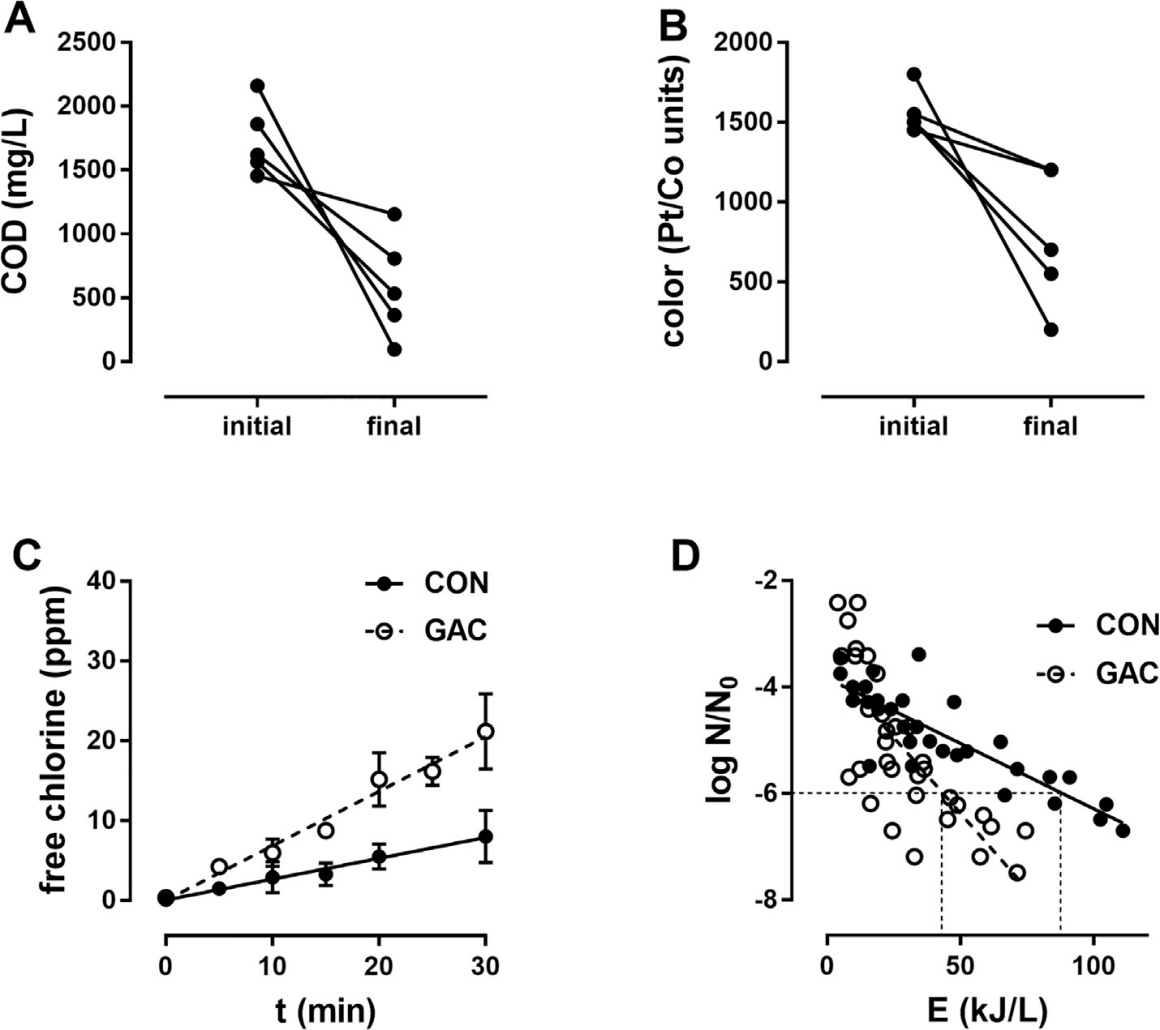
**Summary of bench-scale studies.** Blackwater was run through a 5-L GAC filter in 8-L batches for at least 24 h prior to electrochemical treatment. **A:** Reduction in COD with GAC treatment over 5 experiments, initial and final measurements for each experiment shown **B:** Reduction in color with GAC treatment over the same experiments as in (A), initial and final measurements for each experiment shown. **C:** Free chlorine generated in GAC vs CON (untreated) blackwater during electrochemical treatment in constant voltage mode at 24 VDC Data shown mean ± S.D. from 5 CON and 6 GAC trials. **D:** Log reduction values in MPN versus electrochemical energy (E) from the same trials as the data shown in (**C**). Dotted lines indicate the estimated energy to achieve 6-log reduction based on linear regressions of the data from each group.

To first understand the performance of the GAC filters, 8-L batches of blackwater were circulated through a 5-L GAC filter for at least 24 h. The 8-L batches were subsequently processed with the electrochemical cell to determine disinfection energy required as compared to controls (blackwater from the same batches without GAC treatment). GAC treatment reduced COD from 1732 ± 282 to 590 ± 407 mg/L and color from 1560 ± 134 to 770 ± 432 Pt/Co units prior to electrochemical treatment (Fig. 2A and B). GAC pretreatment also resulted in an increase in the free chlorine production rate from 0.26 ± 0.02 to 0.68 ± 0.05 mg/(L min) (Fig. 2C) and a decrease in energy required to achieve a 6-log unit reduction in bacteria from 88 to 43 kJ/L with the electrochemical process (Fig. 2D). As previously discussed (Hawkins et al., 2018), most of the COD present in these tests would be in the soluble or suspended form as most of the particulate COD is removed in settling tanks preceding these tests. These data support the hypothesis that reducing the soluble COD can reduce the disinfection energy required for an electrochemical process.

### 3.2 Integrated system studies

**Fig. 3:**
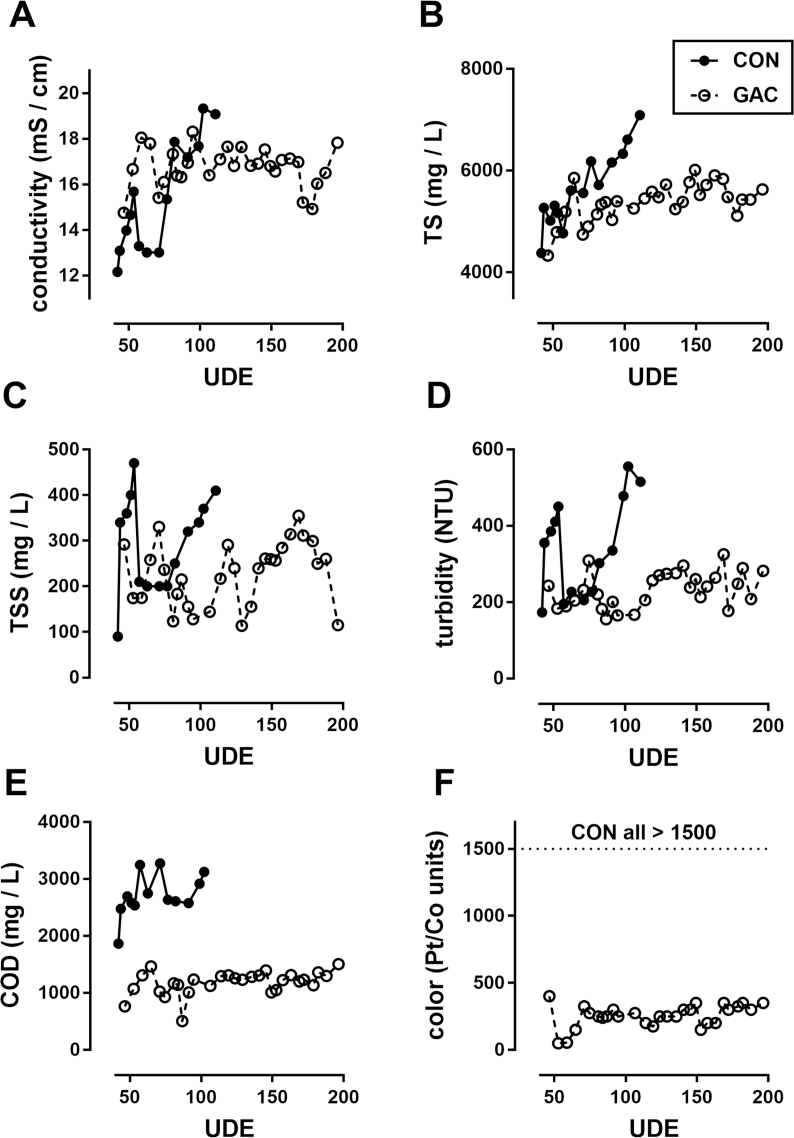
**Performance of integrated GAC filters with repeated treatment and recycling of blackwater.** Shown are data from multiple trials in which blackwater was circulated through GAC filters prior to electrochemical treatment and recycling. Each data point represents a sample taken from a 30-L batch treatment. **A-E:** samples were taken immediately prior to electrochemical treatment; electrochemical treatment had no consistent effect on these parameters. **F**: data from color samples shown were taken following electrochemical treatment; pre-treatment color measurements were typically ∼300 Pt/Co units higher than those shown. (Note: data from CON samples shown for reference in panels **A**, **C**, **D**, and **E** were included in a prior report (Hawkins et al., 2018). Color was not routinely measured in those studies but was consistently well in excess of 1500 Pt/Co units.) UDE = user day equivalents, see Materials and Methods for details.

Based on these positive initial results, the GAC filters were integrated into the operational prototype toilet (Fig. 1B). This was accomplished by circulating the blackwater from the pre-process tank (following the settling tanks) through a pair of GAC filters prior to electrochemical processing, which occurs in 30 L (net) batches. These pre-treatment GAC filters were added to the system after 40 UDE to test performance under steady state conditions. Data from a previous study (Hawkins et al., 2018) conducted in the same system prior to GAC installation were used as control data for comparison. Fig. 3 shows that the addition of the pre-treatment GAC filters had significant impacts on the quality of liquid prior to electrochemical treatment. Most notably, the COD was consistently reduced compared to the previous system run without GAC, and the color was reduced from >1500 Pt/Co units to an average of 228 Pt/Co units. While this meets the targeted discharge standard for color (300 Pt/Co units, Tamil Nadu Pollution Control Board, 2013) the COD remained over 1000 mg/L which is well over the target surface discharge limit of 250 mg/L (Ministry of Environment & Forests, India, 1986).

**Fig. 4:**
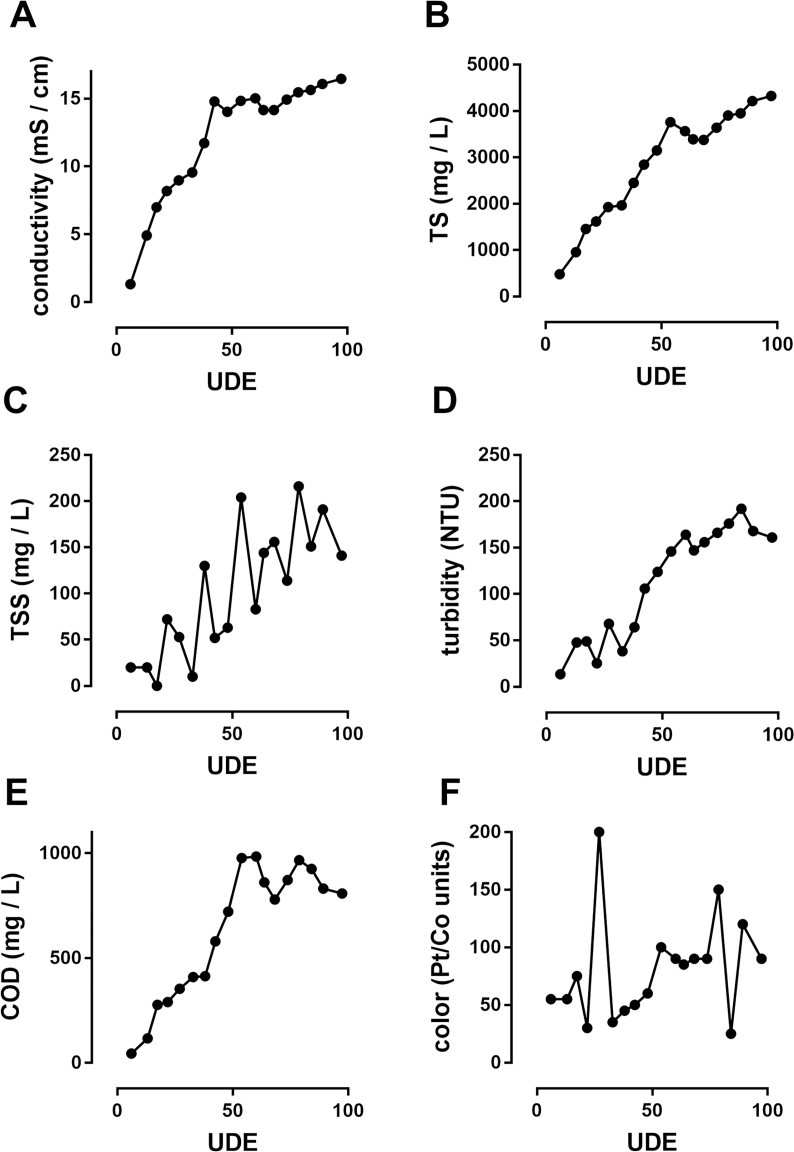
**Startup performance of integrated system with pre- and post-process GAC filters.** Shown are data from multiple trials in which blackwater was circulated through GAC filters both prior to and following electrochemical treatment, and recycled as flush liquid. Samples for all data shown were taken from the process tank at the end of the electrochemical treatment.

In an effort to reach discharge standards, an additional pair of GAC filters were added to circulate the liquid in the holding tank (post electrochemical treatment) prior to reuse for flushing (Fig. 1B). Further, we tested the addition of this “polishing” unit from startup of the system (settling tanks and flush cistern initially charged with tap water). The results of these tests are highlighted in Fig. 4. Comparisons of all parameters in the process tank during the same window of UDE (40–120) among systems without GAC, with pre-process GAC only, and with pre- and post-process GAC units are shown in Table 1. All parameters measured trended downward with the addition of the post-process GAC unit, though the differences were only statistically significant in the cases of conductivity, TS, color, and initial MPN (MPN_0_).

**Table 1:** **Summary of GAC effects on blackwater quality in the integrated system.** Shown are mean ± S.D. (range) values for each parameter indicated in all disinfection trials run between 40 and 120 UDE under each condition, n = 14 (CON and PRE), n = 11 (PRE + POST). *, **, and *** indicate p < 0.05, 0.01, and 0.001, respectively, compared with CON;+and ^+++^ indicate p < 0.05 and 0.001, respectively, compared with PRE. Significance determined by one-way ANOVA with a Tukey's multiple comparison test. Note: data from CON samples shown for reference were included in a prior report (Hawkins et al., 2018). NTU: Nephlometric Turbidity Units; MPN_0_: microbe counts in the liquid coming into the process tank prior to electrochemical disinfection.

	CON	PRE	PRE + POST
conductivity (mS/cm)	15.38 ± 2.45 (12.16–19.33)	16.80 ± 1.01 (14.75–18.31)	15.04 ± 0.79+(14.01–16.44)
total solids (mg/L)	5656 ± 749 (4380–7090)	5171 ± 390 (4332–5858)	3648 ± 447 ***,^+++^ (2843–4346)
total suspended solids (mg/L)	297 ± 107 (90–470)	209 ± 65 * (123–331)	138 ± 55 *** (52–216)
turbidity (NTU)	344 ± 127 (173–555)	208 ± 42 *** (155–309)	155 ± 24 *** (106–192)
COD (mg/L)	2714 ± 374 (1864–3274)	1097 ± 247 *** (504–1464)	845 ± 123 *** (579–983)
color (Pt/Co units)	>1500	228 ± 97 *** (50–400)	86 ± 33 ***, ^+++^ (25–150)
log MPN_0_/ml	6.67 ± 1.22 (3.97–8.04)	4.20 ± 2.43 ** (1.36–8.04)	2.35 ± 0.55 ***,+ (1.63–3.48)

### 3.3 COD removal and disposition

**Fig. 5:**
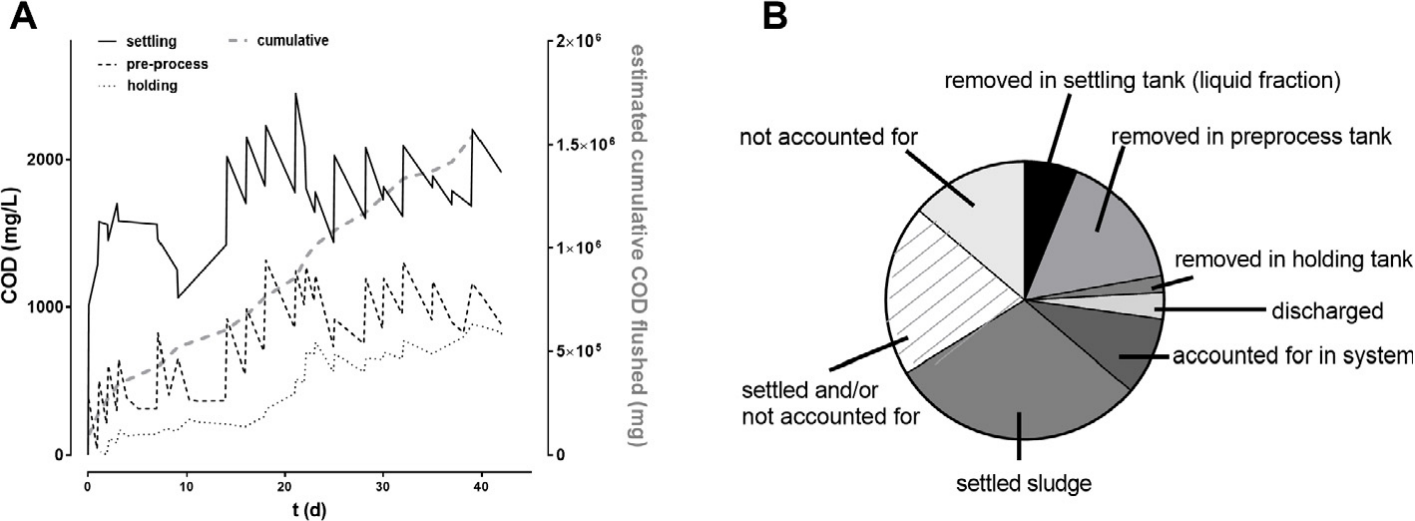
**Tracking COD concentrations throughout the integrated system. A:** COD measurements taken in the settling, pre-process, and post-process holding tanks, respectively (left axis) tracked over seven weeks of continuous testing; dotted grey line indicates the estimated cumulative COD flushed into the system (right axis) over the same time period. Measurements in the settling tanks were taken from the third tank immediately prior to commencing and after finishing flushing. Pre-process tank samples were taken after flushing and before pumping a batch over to the process tank for electrochemical treatment. Holding tank samples were taken immediately after pumping over from the process tank following electrochemical treatment, and prior to commencing flushing with the recycled blackwater. **B**: **Estimated distribution of total COD.** Estimated total removals in the settling, preprocess, and holding tanks were calculated from the removal rates and residence times summarized in Table 2. COD discharged was determined by the volume discharged and the COD measured in the holding tank. COD accounted for in the system was determined by the COD levels measured throughout the system at the end of testing. The total COD flushed into the system and proportion settled into the sludge was estimated as described in Section 3.3.

COD concentrations were tracked in the supernatant fraction of the settling tanks, as well as the pre-process and holding tanks throughout the testing of the system with pre- and post-process GAC units over seven weeks of continuous testing (Fig. 5A). COD removal rates were calculated in each component of the system (Table 2), and the removal rates noted for the GAC filters compare favorably with COD removal rates for GAC previously reported (0.24 ± 0.01 kg_COD_ m_GAC_^−3^ d^−1^, Huggins et al., 2016).

**Table 2:** **COD removal rates in different components of the liquid treatment system.** Data are mean ± S.D. (range).

	COD removal rate	
	mg h^−1^	kg m^−3^_GAC_ d^−1^
settling tanks^a^	195 ± 104 (65–489)	–
pre-process tank GAC	428 ± 197 (101–749)	0.64 ± 0.30 (0.15–1.12)
EC process	58 ± 217 (-340–460)^b^	–
post-process holding tank GAC	64 ± 47 (10–200)	0.10 ± 0.07 (0.01–0.30)

a Data from steady state only (d > 14), see figure.

b Negative values denote that measured COD increased over treatment time.

We estimated the cumulative mass of COD flushed into the system based on the average COD we measured in our pooled urine donations (∼6000 mg/L), the median daily per capita fecal mass and fecal COD reported by Rose (130 g and 71 g, respectively), and the typical removal of solids from our liquid waste stream by our solid-liquid separator (90% of wet weight). Thus:

(3) $$estimated\;cumulative\;COD\left(mg\right)=6000\frac{mg}{L}x\sum v_{urine}+\left(\frac{71,000mg}{130g}x\sum m_{feces}\right)x0.1$$

Using this method, we estimated that 1543 g of COD were flushed into the system in total (Fig. 5A). COD was not routinely measured in the settled sludge beds because of the need to leave these undisturbed for proper function and difficulty of access for sampling; however, we know from periodic measurements (up to 30,000 mg/L COD) that a substantial portion of the COD was associated with particles settled in the sludge beds. Levine et al. (1985) reviewed particle size distributions (PSD) of COD in municipal wastewater streams and found that 34% of the COD was in readily settled particles. Another PSD study by Hocaoglu and Orhon (2013) of blackwater (a more similar waste stream to our own) reported that 62% of the COD was found in particles larger than 1.2 μm. These investigators also looked at the PSD within the settled fraction of their blackwater, and found that the settled fraction included particles significantly smaller than the generally accepted threshold for settling (100 μm), down to 10 μm, likely due to aggregation and co-precipitation with larger particles. Thus, it is reasonable to estimate that between 30 and 50% of the COD flushed into our system remained in the settled sludge.

Using the removal rates in Table 2, we estimated that at least 16% (247 g) of COD was removed by the GAC filters in the pre-process tank, whereas only 2% could be attributed to the GAC filters in the post-treatment holding tank. Given the total amount of GAC in the filters on the pre-process tank was 7.2 kg, this indicates that the adsorption capacity of this material is at least 34 g COD/kg GAC, though it is likely higher given that the filters were still removing COD from the process liquid at the end of the study. This is in agreement with the total COD adsorption capacity of coal-based GAC in wastewater containing 1200 mg/L COD (similar to peak concentrations in our pre-process tank, see Fig. 5A) recently reported at ∼48 g/kg (Huggins et al., 2016). An estimated 6% was removed from the supernatant fraction in the settling tanks, while 9% remained in the liquid system at the end of testing. Most impressively, only 3% of the COD flushed into the system was discharged during regular operation over a seven-week trial period.

Taken together, we can account for 36% of the total COD in the liquid fractions and between 30 and 50% in the settled sludge, leaving between 14 and 34% not readily accounted for (Fig. 5B). Note however that the estimated COD removals by each GAC system are conservative and do not take into account removal that occurred between measurements; while we attempted to cover peak and trough concentrations with the timing of our sampling, there were idle periods between batches where a dead volume (typically 10–15 L of liquid) continued to circulate through the GAC filters. Moreover, peak measurements in the pre-process tank were not taken until flushing was completed, meaning that some of the liquid had already been circulating through the GAC by the time the peak measurement was made. Nonetheless, these data point to opportunities for improving the system. Most notably, given that the greatest removal rate appears to occur in the GAC filters supplied by the pre-process tank, scaling up the media volume in this part of the system would likely effect the greatest increase in COD removal capacity.

At present we do not know what accounts for the consistently differing COD removal rates measured between the pre-process and post-process (holding) tank filters. However, it is possible that most of the fraction of COD readily removed by this media (i.e., the soluble COD) is removed in the pre-process filter, leaving comparatively little to be removed by the unit in the holding tank. It is also possible that the chlorination of the process liquid by electrochemical disinfection affects the adsorptive properties of the GAC (Karanfil, 2006) or prevents formation of a biofilm on the carbon that would contribute to oxidation of COD. Future studies will address the question of whether and how biologically activated carbon contributes to contaminant and nutrient removal in each component of this system. Further work is also required to achieve surface discharge limits for COD and explore nutrient removal efficiency of the system. Ongoing work is exploring the lifetime of the GAC, maintenance requirements, reuse potential of expended media, and the potential of lower-cost substitutes for GAC such as biochar (Huggins et al., 2016), all of which will be critical to define practical implementation.

### 3.4 Impact on energy required for electrochemical disinfection

**Fig. 6:**
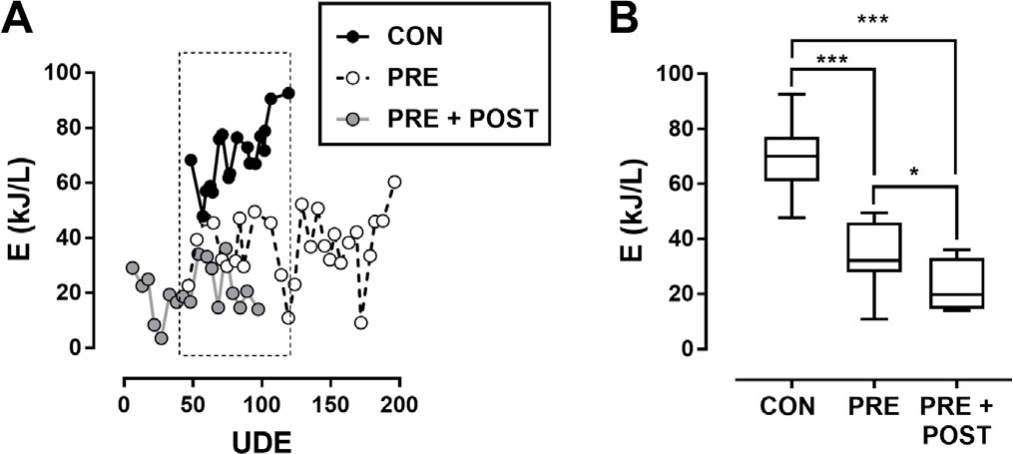
**Effect of GAC on energy required for electrochemical disinfection in recycled blackwater.** Electrochemical energy (E) required to achieve MPN = 5/ml was determined for each 30-L batch process in the integrated system (see Materials and Methods for details.) **A**: E for each trial shown, indexed to UDE. **B**: Box and whisker plots of the data points in the dotted line box in (**A**) to compare trials from the same range of UDE (40–120 UDE). Lines indicate median, boxes 25th and 75th percentiles, error bars maximum and minimum values; * = p < 0.05, *** = p < 0.001 by one-way ANOVA with a Tukey's multiple comparison test for the comparison indicated. Note: data from CON samples shown for reference were included in a prior report (Hawkins et al., 2018).

Fig. 6 shows the energy comparison among all the configurations (control, pre-treatment GAC, and pre- and post-treatment GAC). When compared over the same range of UDE, the median disinfection energy was reduced from 70 kJ/L to 32 kJ/L (54%) with the addition of the pre-treatment GAC filters. An additional improvement to 20 kJ/L was observed with the addition of the post-treatment GAC filters, representing an overall 71% reduction of the energy required for complete electrochemical disinfection of blackwater (MPN < 5/ml) with the integration of GAC filters into the system. As mentioned above, this large improvement in the energy requirement is most likely due to the adsorption of COD by the GAC.

After positive results were observed with the GAC filters in the lab, field versions of both the pre- and post-treatment GAC filters were installed at our field test site at CEPT University in Ahmedabad, India in June 2017. Early qualitative results indicated that users were pleased with the visual appearance of the recycled liquid and reported little to no odor (Elledge et al., unpublished results.)

## 4 Conclusions

• A hybrid system integrating both the pre- and post-treatment GAC filters with electrochemical treatment resulted in substantial reduction in the steady state concentrations of several contaminants compared to the same system without GAC. Most notably, 1) the COD of the process liquid was reduced by 69%, and 2) the appearance of the water was greatly improved indicated by the reductions in turbidity and color.
• The energy required for complete disinfection was reduced by 71% to 20 kJ/L with the addition of both pre- and post-treatment GAC filters. This energy reduction is most likely due to the reduction in specifically soluble COD (Hawkins et al., 2018).

## Author contributions statement

This study was conceived by Tate Rogers, Katelyn Sellgren, and Brian Hawkins. Tess Rogers, Mikayla Stoner, and Brendon Lynch performed the data collection. Tate Rogers, Aaron Forbis-Stokes and Brian Stoner provided input on study design and data analysis. Brian Hawkins designed the study, oversaw data collection, and performed the data analysis. The manuscript was written by Tate Rogers and Brian Hawkins. All authors provided comments on the manuscript prior to its submission.
